# Host–Parasite Battles Shed Light on the Evolution of Gene Expression

**DOI:** 10.1371/journal.pbio.0030252

**Published:** 2005-05-31

In human–pathogen encounters, the battle for advantage plays out at the level of gene expression. Hosts will stand a better chance of surviving if their genes confer resistance to diverse pathogens, while pathogens need genes that promote virulence and infection. To understand this game of evolutionary one-upsmanship, biologists study the genetic basis of resistance and infection by investigating how changes in an organism's genetic makeup, or genotype, affect its physiological and physical makeup, or phenotype.

In a new study, Scott Nuismer and Sarah Otto detail how host–parasite interactions shape the changes in gene expression that alter an organism's ability to induce or resist infection. They find that gene expression for host and parasite follows quite different evolutionary paths: hosts express as many different gene variants, or alleles, as possible, while parasites express very few alleles. It's in the host's interest to have as many genetic weapons as possible that can recognize a foreign invader, while it's in the parasite's interest to reduce the number of recognizable molecules for a host to latch on to and destroy. Even though these results are intuitive, this phenomenon has not been shown before.

To model the evolution of gene expression levels in a host–parasite interaction, Nuismer and Otto started with a single gene A in hosts and a single gene B in parasites with two alleles each (*A* or *a*, and *B* or *b*) that are involved respectively in resistance in the host and promoting infection by the parasite. Nuismer and Otto's model allows gene expression levels to be regulated by an additional modifier gene in hosts and parasites. The two variants of the modifier gene (*M* and *m*) alter the expression of the host gene A or parasite gene B. As a result of host–parasite interactions, the alleles at the modifier gene either evolve to increase expression of only one allele or to co-express both alleles. [Fig pbio-0030252-g001]

**Figure pbio-0030252-g001:**
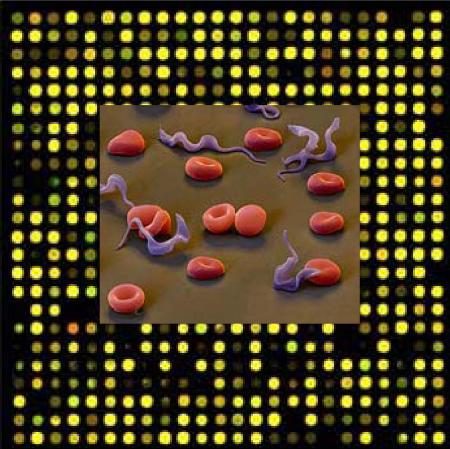
Interactions between hosts and parasites shape changes in gene expression, potentially maximizing parasite recognition for hosts while minimizing detection for parasites (such as red blood cells and trypanosomes)

When parasite and host are allowed to interact, the model shows that host resistance alleles typically evolve toward co-expression while parasite infection alleles evolve toward single expression. By expressing more than one gene at a time, the host can recognize a greater diversity of parasites. But what's good for the host is bad for the parasite. Hosts benefit from a wider array of parasite recognition systems, while parasites benefit from expressing a narrow range of antigens to evade the host recognition system.

Human immune cells, for example, can recognize billions of different antigens, which then triggers an immune response against the foreign substance and increases the chance of surviving the infection. Parasites, however, generally express only one of many possible antigen alleles. The parasite responsible for African sleeping sickness expresses only one of thousands of surface receptor genes, which offers the host fewer opportunities for detection.

Nuismer and Otto's model provides a framework for understanding empirical observations of allele expression in known host–parasite interactions and may well help explain similar modifications in allele expression in other systems. Because the model also provides testable predictions, it should be useful in interpreting data from a wider range of species and interactions, furthering our understanding of the evolutionary forces that shape infection and resistance and ultimately influence how genes evolve.

